# From Temple to Emergency Room: A Chemical Injury Case Report

**DOI:** 10.7759/cureus.63851

**Published:** 2024-07-04

**Authors:** Apurva Prabhudesai, Abhay Lune

**Affiliations:** 1 Ophthalmology, Dr. D. Y. Patil Medical College, Hospital and Research Centre, Dr. D. Y. Patil Vidyapeeth (Deemed to be University), Pune, IND

**Keywords:** vision, ocular emergency, corneal injury, calotropis, chemical injury

## Abstract

*Calotropis* is a small perennial plant that is native to regions with tropical climates in countries like India, where it is found throughout the country. The leaves of the* Calotropis* plant have been used as an offering to gods since ancient times. Accidental contact with the sap of the plant, called latex, can lead to eye injury and affect vision significantly if left untreated. However, if treated in time and appropriately, vision can be restored. A 30-year-old gentleman reported to emergency medicine with accidental contact in his right eye with* Calotropis* plant sap. He had complaints of blurring of vision, foreign body sensation, and intolerance to light. On ocular examination, there was conjunctival congestion with corneal edema with Descemet's membrane folds. The best-corrected visual acuity (BCVA) in the right eye was 6/18 parts not improving on the pinhole. The patient was started on systemic and topical antibiotics, topical steroids, and lubricating drops immediately. After two months of treatment, the vision improved gradually, the BCVA in the right eye was 6/6, and the patient was asymptomatic. This is a case report of an uncommon injury due to plant sap, with grave consequences if left untreated. Early intervention and prompt medical management led to recovery in a short time period.

## Introduction

*Calotropis* is a medium-height perennial shrub found in open drylands. The plant contains cardenolide and proceragenin, the leaves and stalk contain calotropin and calotropagenin, the flower contains calotropenyl acetate and multiflavenol, and the latex contains uzarigenin and terpenol ester [1]. Very few cases of *Calotropis* chemical injury have been reported. This can be because these injuries mostly occur in rural regions as compared to urban areas. People who come into contact with the sap of the *Calotropis* plant usually resort to the use of the latex of this plant as a locally applicable Ayurvedic medicine [2]. Accidental contact with the sap of the *Calotropis* plant can affect vision and have ocular manifestations like corneal edema, superficial punctate epitheliopathy, and Descemet's membrane folds [3].

## Case presentation

**Figure 1:**
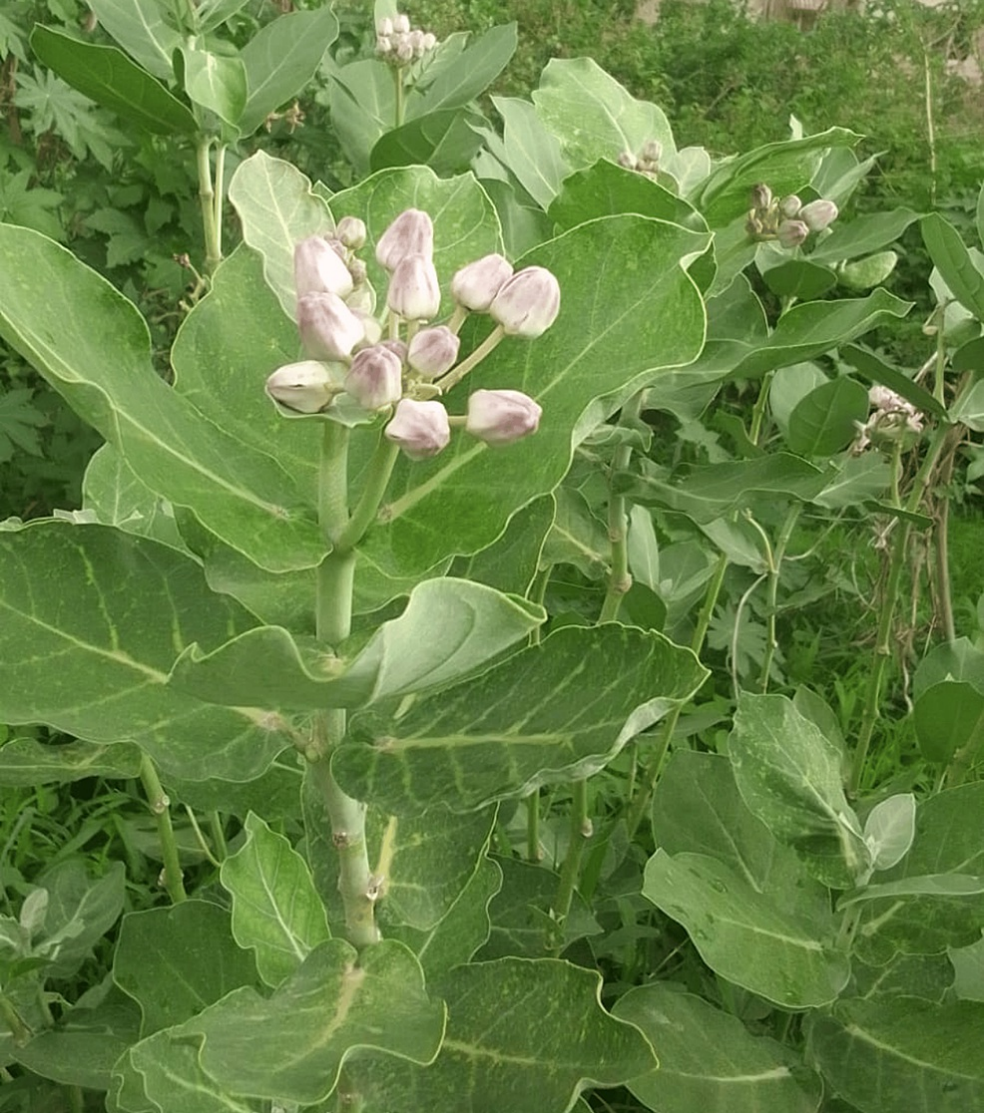
Calotropis plant as seen in the wild. The leaves and flowers of the plant are offered to local deities (original image from the author).

A 30-year-old gentleman reported to the emergency medicine department with complaints of blurring of vision, pain, foreign body sensation, redness, and intolerance to light in the right eye for one day. The patient gave a history of accidental eye contact with the sap of the *Calotropis* plant (Figure 1) while he was offering it to a local deity.

There was no history of itching or discharge present. There was no history of any systemic illness. Ocular examination on presentation in the emergency room is presented in Table 1.

**Table 1:** Ocular examination on presentation in the emergency room

	Right eye	Left eye
Vision	6/18, N12	6/6, N6
Pinhole vision	6/12, N 12	6/6, N6
Conjunctiva	Circum-ciliary congestion	Normal
Cornea	Corneal edema with Descemet’s folds all over Superficial punctate epitheliopathy inferiorly	Clear
Anterior chamber	Normal depth	Normal depth
Iris	Normal pattern	Normal pattern
Pupil	Central, circular, reacting to light	Central, circular, reacting to light
Lens	Hazily seen, clear	Clear
Fundus	Hazily seen, optic disc and macula appear normal	Optic disc and macula normal

**Figure 2:**
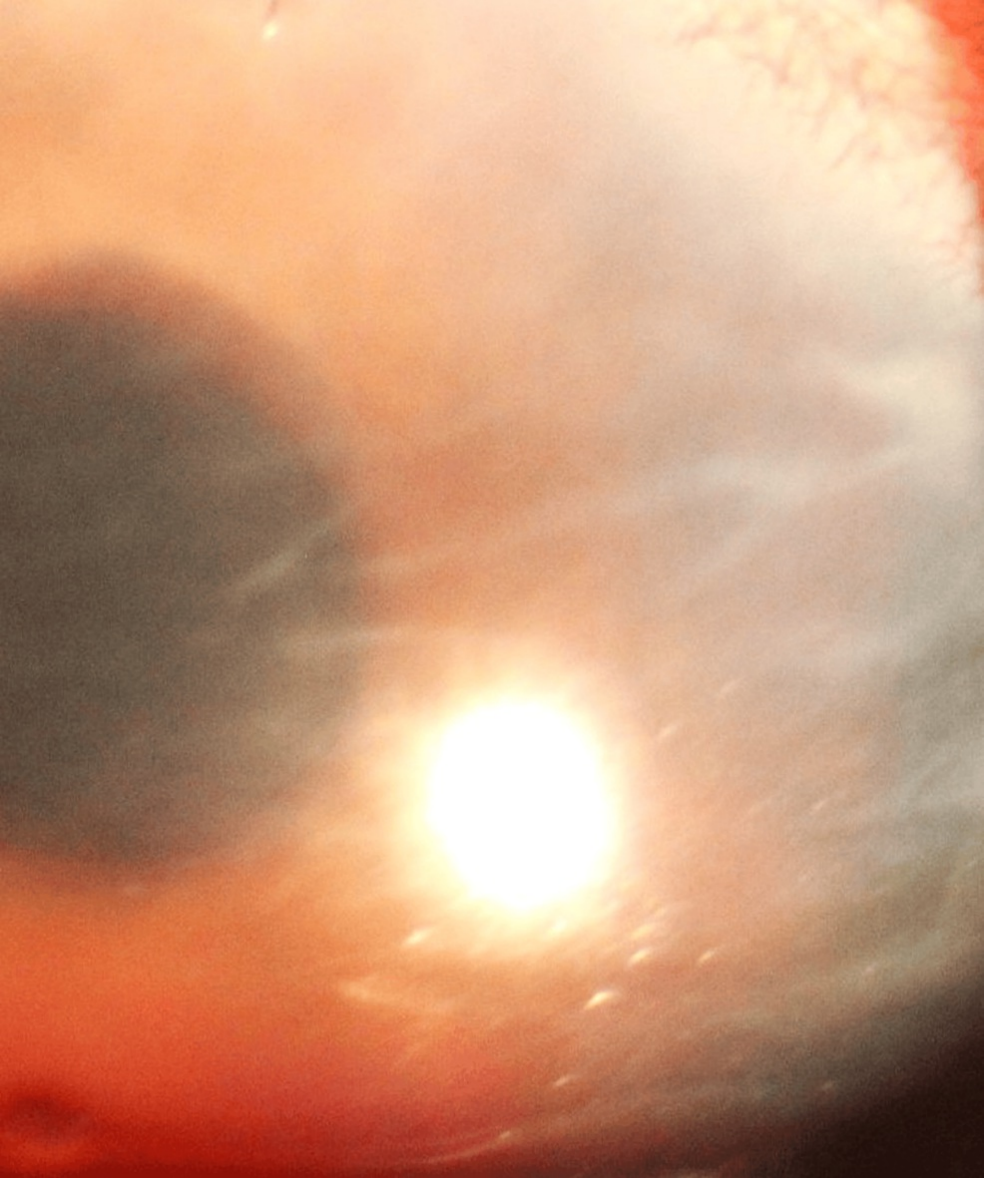
Slit-lamp image of the Descemet's membrane folds (original image from the author)

In the right eye, the uncorrected visual acuity was 6/18, and with the pinhole, it improved to 6/12p. The left eye vision was 6/6. On the anterior segment examination of the right eye on a slit-lamp microscope, the conjunctiva had circum-ciliary congestion, and the cornea showed Descemet membrane folds (Figure 2), present all over with superficial punctate epitheliopathy present inferiorly. The anterior chamber did not show any presence of cells or flare. Intraocular pressure (taken digitally by the examiner with her two index fingers in the emergency room) was normal. The left eye examination did not reveal any abnormality. Fundus examination of the right eye had hazy media, the optic disc was normal, the cup:disc ratio was 0.3, the NRR was healthy, the macula was normal, the fundus reflex was present, and the general fundus was within normal limits. The left eye fundus examination did not reveal any abnormality.

The patient was started on tablet doxycycline 100 mg BD for three days, eye drops prednisolone 1% TDS, moxifloxacin 0.5% QID, homatropine 2% BD, and lubricating eyedrops two hourly. On day 1, there was no improvement in the visual acuity of the right eye. The anterior segment examination did not reveal any change from the previous examination.

The same medications were continued. Intra-ocular pressure was checked on noncontact tonometry. In the right eye, it was 20 mm Hg, and in the left eye, it was 16 mm Hg. In the right eye, timolol 0.5% eye drops BD and a lubricating ointment at night were started. 

On day 3, the visual acuity of the right eye improved from 6/18 to 6/9p with the pinhole. On the anterior segment examination, circum-ciliary congestion was present with no change in degree, Descemet's membrane folds were seen to be reduced, and the pupil was pharmacologically 5 mm dilated, not reacting to light. The patient was symptomatically better.

At follow-up on the seventh day, the visual acuity of the right eye improved to 6/12. Circum-ciliary congestion was reduced along with a reduction in the corneal edema and Descemet's membrane folds. The intra-ocular pressure was 18 mm Hg on the noncontact tonometry in the right eye. Topical antibiotics were stopped. Topical steroids were tapered every five days. The patient was asked to stop homatropine after one week.

At the one-month follow-up, the patient's right eye vision was 6/9, N8, and the conjunctiva and cornea were clear with a quiet anterior segment. The intra-ocular pressure was 16 mm Hg on noncontact tonometry in both eyes. At the two-month follow-up, the patient’s right eye vision was 6/6P, N6. The RE cornea was clear, and the anterior segment was quiet.

## Discussion

*Calotropis* (madar, akdo) is a plant found throughout India. It has medicinal properties and has a cultural significance as the flowers are offered to many Hindu deities. *Calotropis procera* generally grows in desert areas. It is also commonly called milkweed due to the characteristic latex production of the plant. The milky sap or latex produced by the plant consists of a compound named calotropin, which is toxic in nature and has strong ocular cytotoxic properties [3].

*Calotropis* causes ocular manifestations due to the acidic nature of latex and due to toxins. *Calotropis* latex is capable of penetrating the corneal stroma and can cause permanent damage to the corneal endothelial cells. Ocular manifestations have been divided into two stages [4]: (1) stage of acute acid injury, which is the immediate cause of the burning sensation, pain, and photophobia, and (2) stage of toxicity, which is seen after a few hours. There is a diminution of vision, which is due to corneal defects. The two prominent features of toxicity due to *Calotropis* sap are corneal edema and Descemet’s membrane folds.

Unlike other chemical burns, *Calotropis* latex is nontoxic to the corneal epithelium but significantly toxic to the corneal endothelium [5]. Our patient reported within eight hours after injury with moderate vision loss, pain, and photophobia. 

In a study by Kumar et al., most of the patients presented a few hours after the injury. This was due to a lack of significant pain. Delayed diminution of vision over a period of two to four hours was a common finding in all cases. There was no pain after the initial burning sensation. Most of the cases had inferior conjunctival staining and corneal edema with Descmet’s membrane folds [6]. 

In a study by Huda et al., the composition of active compounds in the *Calotropis* latex was analyzed with the help of phytochemical screening. It was found that the latex contains several alkaloids (calotropin, catotoxin, calcilin, and gigantin), which are poisonous in nature. Moreover, the pH value was 4.2 at room temperature of 25 °C. It was concluded that the stromal keratitis was due to the inflammation induced by the strong pro-inflammatory properties of these toxins. The use of local corticosteroids helps in the resolution of keratitis and supports this theory [7].

In our case, the patient was treated with topical steroids, antibiotics, and cycloplegics, and he recovered within three days. In a case study by Waikar and Srivastava, the patient was treated in a similar manner and recovered in two days. Conjunctival staining and Descmete's membrane folds persisted for two more days [8].

In a study by Pandey and Sahu, 25% of the patients had epithelial defects. Stromal edema and Descemet’s folds were persistent when steroids were withheld in patients with epithelial defects. They started resolving once topical steroids were started. It was concluded that striate keratitis resolves with topical steroids [9].

In a retrospective cross-sectional hospital-based study done by Varsha et al. in a period of 10 years, 2,047,360 new patients were included, and out of these, 362 patients (incidence of 0.018%) were diagnosed with *Calotropis* poisoning during the study period. The most common findings were Descemet’s membrane folds in 57.35%, followed by stromal edema in 30.39%, epithelial defects in 24.51%, and superficial punctate keratitis in 25% of patients. Patients with *Calotropis* poisoning were mainly managed with medical treatment. This consisted of steroid eyedrops, lubricants, antibiotic eyedrops, and mydriatics. Rarely, the surgical treatment was required in the form of an amniotic membrane graft (0.8%) [10].

## Conclusions

Offering of *Calotropis* leaves and flowers to local gods is a common practice in some areas. Awareness is necessary about how the milky sap can affect the vision if it comes in contact with the eyes. People should also be told about basic first aid, not to self-medicate, and to go to the nearest hospital if there is any accidental contact.

Early intervention and prompt management show promising results in the form of full recovery of corneal edema and visual acuity, in a short period of time. Simple health education in the form of hand washing and avoiding eye contact and eye rubbing while plucking the flowers and leaves of *Calotropis* can prevent this injury.
